# Endoscopic endonasal resection of a large recurrent tuberculum sellae meningioma with vascular adherence

**DOI:** 10.3171/2025.10.FOCVID25148

**Published:** 2026-01-01

**Authors:** Ashutosh Carpenter, Jaskaran S. Gosal, Akhilesh Gowda G. Basappa, Syed Yasin S. S. Emanee, Pooja Choudhary, Easwer H. Venkat, Krishnakumar Kesavapisharady, Gowtham Matham, Prakash Nair

**Affiliations:** 1 Department of Neurosurgery, Sree Chitra Tirunal Institute for Medical Sciences and Technology, Thiruvananthapuram, Kerala, India; and; 2 Department of Neurosurgery, All India Institute of Medical Sciences, Jodhpur, Rajasthan, India

**Keywords:** endoscopic endonasal resection, tuberculum sellae meningioma, recurrent ACF meningiomas, meningiomas with vascular involvement

## Abstract

Tuberculum sellae meningiomas often compress the optic apparatus and encase the ACA-ACom complex, making approach selection challenging. This video demonstrates endoscopic endonasal resection of a large, recurrent, tuberculum sellae meningioma with severe adherence to the ACA-ACom complex. A transplanum-transtuberculum corridor provided direct access to the dural base, enabling early tumor devascularization and meticulous dissection of microvasculature to the optic nerves, pituitary stalk, and hypothalamus. Unroofing the optic canal facilitated early optic nerve decompression, reducing the risk of neural injury during tumor manipulation. Multilayer skull base reconstruction achieved watertight closure. Gross-total resection was achieved without ischemic sequelae, and postoperative visual function improved significantly.

The video can be found here: https://stream.cadmore.media/r10.3171/2025.10.FOCVID25148

## Transcript

This video demonstrates the endoscopic endonasal resection of a large recurrent tuberculum sellae meningioma with vascular adherence to the ACA-ACom complex.

### 0:30 Clinical History and Examination.

A 67-year-old female patient presented to us with progressive deterioration of vision for a period of 2 years. She had undergone cataract surgery in her left eye with no improvement in vision. On examination, she had only perception of light in her left eye and she could count to 2 feet with her right eye. Visual field testing also showed severely constricted visual fields in both her eyes.

She gave history of a prior endoscopic endonasal procedure for a skull base tumor. However, no medical records or histopathology was available.

### 1:01 Radiological Imaging: MRI and CT Findings.

Contrast-enhanced MRI shows a very brightly enhancing tumor occupying the suprasellar cistern. The inferior extension into the sphenoid sinus reaches up to the anterior aspect of the pituitary gland. The imaging features are diagnostic of a tuberculum sellae meningioma. The pituitary stalk marked by the red arrow can be seen pushed posteriorly. When we look at the blood vessels, the anterior communicating artery complex, especially the right A1, seems to be running within the tumor.

The CT scan shows a very large, hyperdense tumor centered around the tuberculum. However, the extent of bone removal during the prior procedure seems to be extremely limited. This gives the surgeon a very limited amount of access to the anterior aspect of the tumor, which can be seen colored in red.

### 1:48 Preoperative Plan: Bone Removal and Exposure to Enable Early ACA–ACom Control.

Adequate bone removal of the tuberculum and the anterior aspect of the planum, including bone over the optic nerves, is essential for giving access to the anterior pole of the tumor and the entire base of the tumor.^1^ This allows adequate vascular control of the ACA-ACom complex during surgery.

### 2:08 Planned Approach Rationale: Extended Endoscopic Endonasal Approach Versus Transcranial Approach—Advantages and Disadvantages.

The extended endoscopic endonasal approach exploits a direct midline corridor and avoids any brain retraction.^2^ A tailored bone removal permits early exposure of the optic nerves and also early visualization of the ACA-ACom complex.^3^ The devascularization of the dural base and the visualization of the suprasellar cistern are often unmatched with this approach. This occurs with minimal amount of cortical manipulation, which translates to an overall decreased rate of morbidity and consistently superior visual outcomes.^4,5^

Its limitations include a higher risk of postoperative CSF leak and associated complications like meningitis and ventriculitis. This is a major limitation of this approach and requires meticulous skull base reconstruction. The lateral extent of the approach is restricted by the optic nerves, rendering it unsuitable for lesions with wide en plaque dural base or extension beyond the optic nerves or the ICA. Management of vascular adherence to the ICA or the ACom complex demands a greater degree of technical skill and has a steep learning curve.

Transcranial approaches in contrast provide broad lateral exposure, ideal for lesions with extensive lateral or en plaque spread, and gives better control over vessels when the tumor is attached to it. The approach also has a lower incidence of postoperative CSF leak rates.

These advantages, however, need to be balanced with the need for brain retraction and the attendant risks of edema and contusions.^6^ There is also a very limited amount of exposure to the inferomedial aspect of the optic nerve and the sellar floor.^3,7^ Neurovascular manipulation of the optic nerve has a greater risk of resulting in ipsilateral visual deterioration. Other potential complications include anosmia, seizures, and cosmetic concerns such as a craniotomy scar or temporalis atrophy.

### 3:55 Surgical Procedure: Inspection and Elevation of Nasoseptal Flap.

The initial nasal inspection reveals a clean nasal cavity with no synechiae. A wider nasoseptal pedicle is seen on the left side. Hence, we elect to elevate the nasoseptal flap on the left side. The incision is taken along the floor of the nasal cavity to elevate a very wide nasoseptal flap. This has to be done very carefully because of the presence of a sharp septal spur on the left. The flap is elevated in a submucoperiosteal fashion and stored in the choana for use later on during surgical reconstruction of the skull base.

### 4:30 Unroofing of Optic Nerves and Tumor Exposure.

Once the flap has been elevated, the anterior cranial fossa is drilled to decompress both the optic nerves. Unroofing the optic nerves minimizes the amount of ischemia to the optic nerve during surgical manipulation of the tumor.

### 4:44 Arachnoid-Preserving Debulking With Circumferential Neurovascular Dissection.

An extremely fibrous tumor is encountered during surgical dissection. This is extended into the sphenoid sinus. We use a Cavitron ultrasonic aspirator for internal decompression of the tumor, which is then followed by circumferential mobilization of the tumor away from the adjacent neurovascular structures. Maintain the arachnoidal plane while we dissect the tumor, and here we can appreciate the pituitary stalk that lies posteriorly.

We now turn our attention to the left optic nerve and the internal carotid artery. One can appreciate these fibrous attachments between the tumor capsule and the ICA. The superior hypophyseal artery can also be seen here in the surgical field. These fibrous attachments are probably the result of a prior surgical procedure, and they are separated and cut using sharp microscissors. As we progress posteriorly, one can start to see the PCom and this is also slowly moved away from the capsule of the tumor. Small parts of the tumor are resected and the tumor is continuously debulked. As we turn our attention to the anterior pole, we see these attachments between the branches of the right A1, the olfactory nerve, the gyrus rectus, and the right optic nerve.

The arachnoidal plane is respected and the tumor needs to be continuously debulked while it is being manipulated. These attachments from the right optic nerve are being slowly separated as the tumor is mobilized. On the left side, we can visualize the left A1 and the A2.

Microsurgical dissection of A2 is required to move it away from the tumor capsule. Inadvertent traction upon the tumor can cause avulsion of these small branches that arise from the A2. Instead, meticulous dissection of the arachnoid can be done to move it away from the tumor.

Arachnoidal attachments are being slowly stripped away from the anterior surface of the tumor capsule using the sharp end of a microdissector. This plane can be developed till we reach the anterior interhemispheric fissure. At this point of time, the upper pole of the tumor can be seen in close relationship with both the A2s.

As we dissect the arachnoidal attachments at the upper pole of the tumor, we start to notice very tenacious attachments between the right A1, the vascular takeoff of the recurrent artery, and the ACom complex. We place a small sharp incision on the tumor capsule and a thin bit of tumor is left along the vessel. This plane is again developed using the sharp end of a microsurgical dissector.

The sleeve of tumor left on the vessel wall prevents avulsion of the vessel as we manipulate the tumor. The plane is developed and using sweeping movements of the microsurgical dissector, the attachments are slowly separated as the tumor lying posterior to the ACom complex is rolled into the surgical field. Sharp dissection to cut these tumor attachments releases the entire tumor, which is then removed from the surgical field.

### 8:24 Postresection Neuroanatomical Survey.

Once this tumor has been delivered, one can start to appreciate the interpeduncular cistern and the suprasellar cistern along with both the optic nerves, the pituitary stalk, the anterior aspect of the third ventricle, the basilar bifurcation, and the oculomotor nerves. A thin rind of tumor attached to the right A2 can also be appreciated.

### 8:46 Multilayer Skull Base Reconstruction.

After hemostasis, we perform reconstruction in a multilayer fashion. A first layer of inlay collagen is then supported by an onlay of facia lata and fat, which is used to seal the skull base using an autologous gasket seal technique.^8^ This repair is then further augmented using a nasoseptal flap.^9^

In this video, we have shown how tenacious vascular attachments between a tuberculum sellae meningioma and the ACom complex can be dissected safely.

### 9:22 Postoperative Course.

This patient made uneventful surgical recovery. She had lumbar drainage for 3 days and she was discharged 5 days following surgery. She had subjective improvement in the vision of her right eye, and at 3 months visual field examination showed clearance of the nasal field cuts that were present before surgery. The biopsy of this tumor was a meningothelial meningioma. At 2 years, she has no recurrence of tumor. The MR imaging showed no new vascular infarcts. A thin rim of stable enhancement without any progression over the past 2 years has been noted close to the ACA-ACom complex.

### 10:06 References.

Thank you.

## Disclosures

The authors report no conflict of interest concerning the materials or methods used in this study or the findings specified in this publication.

## Author Contributions

Primary surgeon: Nair. Assistant surgeon: Basappa, Matham. Editing and drafting the video and abstract: Nair, Carpenter, Gosal, Emanee, Choudhary, Venkat, Matham. Critically revising the work: Nair, Gosal, Emanee, Venkat, Kesavapisharady, Matham. Reviewed submitted version of the work: Nair, Gosal, Basappa, Kesavapisharady. Approved the final version of the work on behalf of all authors: Nair. Supervision: Venkat. Edited anatomical images in the video: Gosal.

## Supplemental Information

### Patient Informed Consent

The necessary patient informed consent was obtained in this study.
